# Grain of Corn in the Windpipe

**Published:** 1889-12

**Authors:** E. W. Row

**Affiliations:** Orange, Va.; Ex President Medical Society of Virginia


### GRAIN OF CORN IN THE WINDPIPE—TRACHEOTOMY —SUCCESSFUL.

BY E. W. ROW, M. D., OF ORANGE, VA.
EX PRESIDENT MEDICAL SOCIETY OF VIRGINIA.

The son of C. C. Y., age 4 years, refreshed himself on Saturday, October 5, 1889, with parched corn, and accidentiy drew one of the grains into the windpipe. Violent cough and dyspnœa immediately ensued. Drs. C. M. Moncure and C. C. Conway saw the boy and administered agomorphia hypodermically, which was followed by free emesis, the stomach ejecting its entire contents. Among the dejected mass were found several grains of corn. The relaxation produced by the emetic, caused more comfortable breathing, and it was thought by his attending physicians that the trouble had been removed.

In a few hours, however, the threatening symptoms returned. and Dr. Moncure was again called. He administered ipecac, producing again, by the relaxation it caused, easier breathing and then followed sleep.

At 12 o'clock at night I was called to see the little sufferer, and found him in an unquiet slumber. The muscus rale could be heard several feet from the patient, and upon applying fingers to the trachea the foreign substance could be felt as it moved to and fro in respiration. He had high fever, a frequent, quick and corded pulse, flushed face, and respiration was hurried and irregular. In a few moments the child awoke suddenly, with short muffled cough and all the symptoms of impending suffocation—which was, to a very great extent, soon ameliorated by hot water and mustard plaster applied over the throat and inhalation of warm vapor from hot water, and he was given a powder composed of hydrarg. chloridi mite, gr. j; atropiæ sulphas, gr. $\frac{1}{60}$, and pulv. ipecac, gr. j. The patient again slept, and in an hour the fever abated to a considerable degree. The pulse became soft and less frequent, and the breathing easier than (the father said) it had been since the accident occurred. About 2 o'clock A. M., I left the patient, directing that I be called if the least change took place in respect to increased difficulty in breathing.

On arriving at my office I collected the surgical instruments necessary to perform tracheotomy and placed them in my case to have handy, should the symptoms become threatening enough to require the operation before daylight.

About 7 A. M. the father came hastily for me, stating that he "feared the boy would be dead before I could get to him," (a distance of little over 100 yards.) When I arrived the little fellow was unconscious; his face was black with venous blood; yet his head was tossing from side to side, and he had a sort of muffled, suffocative cough and looked as though each gasp would be his last. I took the bistoury for immediate operation; but the struggles were so great, and with no one collected enough to assist, I concluded to give chloroform cautiously, with a view of facilitating and rendering sufficient insensibility for a successful operation. To my great relief the chloroform calmed the convulsive cough, inspiration became fuller, and expiration less impeded; the dark blood soon began to leave the face, and finally he began to breathe so quietly that I felt confident that the operation could be delayed until some assistance could be obtained.

Dr. C. M. Moncure was sent for and came promptly, and concurred with me as to the necessity of an operation. He rendered me great assistance during the operation. Tracheotomy was performed, and a large grain of corn taken out of the trachea. After respiration became well established *per vias naturales*, and the bleeding had ceased, the wound was closed by adhesive strips, and recovery rapidly occurred, without an untoward symptom.

In reporting this case, I am aware that it is not of much interest to the profession, but it served to refresh my recollection of a similar case, which I attended in connection with Dr. C. W. Hume about thirty years ago, in which operative interfererence was not permitted by the family, yet the little patient continued to live, although he became reduced to almost a skeleton. But finally he was relieved by nature and abcesses having formed and ruptured into the bronchus, washing out the grain of corn. In that case the outer hard portion of the grain was intact, while the heart or mealy portion had been absorbed. This patient, after daily expectation of death, for more than a month, recovered, and may be seen almost any court day at Orange C. H., the finest specimen of mature manhood, measuring six feet and weighing two hundred pounds.

In all cases where foreign bodies are suspected in the air passages, notwithstanding the dyspnœa and threatened asphyxia may, for the time, be partially relieved by relaxing remedies, yet, it is the imperative duty of the surgeon to watch the case closely, and to apprise the patient's friends of tee probable alternative, so that both he and they should be fully prepared if finally operative interference is demanded.

With respect to diagnosis of bodies in the windpipe, I believe, when it is not impacted, the foreign substance can be easily felt in the treachea as it passes along that tube in the up and down movement caused by respiration; and I believe, also, (but I may be mistaken) that a foreign body may be detected by auscultation, by the rapidity of the movement and suddenness of the "thud" (so to speak) when compared with the movement of mucus in the tubes. The impression of the latter upon the ear is more diffused, does not start so suddenly, pass so quickly, or stop so abruptly, and without a distinct "thud" before reversing its motion.

I would also suggest that coloroform is not contra-indicated in partial obstruction by a foreign body in the air passages, where a great factor in the production of symptoms threatening asphyxia, etc., is dependent upon spasmodic muscular contraction.—*Va. Med. Mo.*

